# SJMHE1 protects against excessive iodine-induced pyroptosis in human thyroid follicular epithelial cells through a toll-like receptor 2-dependent pathway

**DOI:** 10.7150/ijms.66167

**Published:** 2022-03-21

**Authors:** Zhu Zhang, Jiameng Liu, Chaoming Mao, Shan Zhang, Xuefeng Wang, Liyang Dong

**Affiliations:** 1 Department of Nuclear Medicine, The Affiliated Hospital of Jiangsu University, Zhenjiang, Jiangsu, 212000, P. R. China.; 2 Center for Clinical Laboratories, The Affiliated Hospital of Guizhou Medical University, Guiyang, Guizhou, 212000, P. R. China.

**Keywords:** Schistosoma japonicum peptide (SJMHE1), Hashimoto's thyroiditis, Pyroptosis, Toll-like receptor 2 (TLR2)

## Abstract

To elucidate the effect of Schistosoma japonicum peptide (SJMHE1) on pyroptosis in thyroid follicular epithelial cells (TFCs) induced by excessive iodine and the potential mechanism, the effects of SJMHE1 were investigated in NaI-treated Nthy-ori 3-1 cells; and the involvement of the ROS/MAPK/NF-κB signaling pathways in these effects was evaluated by employing CCK-8 assays, flow cytometry, ELISA, and Western blotting experiments. We found that SJMHE1 significantly reduced NLRP3, N-terminus of gasdermin D (GSDMD-N) and cleaved caspase-1 (C-caspase-1) expression, and decreased IL-1β secretion in TFCs. SJMHE1 also markedly reduced reactive oxygen species (ROS) production, and decreased the phosphorylation levels of MAPK and NF-κB pathway members. Moreover, blocking of the Toll-like receptor 2 significantly impaired SJMHE1-mediated protection from excessive iodine-induced pyroptosis in TFCs. Therefore, our results suggested a protective role of SJMHE1 in excessive iodine-induced pyroptosis in TFCs, which might be attributed to its suppression for ROS/MAPK/NF-κB signaling pathway by binding of SJMHE1 with TLR2.

## Introduction

Hashimoto's thyroiditis (HT), the most common organ-specific autoimmune disease 1, which is characterized by the production of thyroid-specific autoantibodies, mononuclear cell infiltration and destruction of the thyroid, resulting in the development of hypothyroidism 2. The incidence of HT has rapidly increased in recent years and is higher in condition of iodine-sufficient with respect to the deficient one 3, 4. Currently, the focus of managing autoimmune hypothyroidism has been on thyroxine replacement 5, however, replaced thyroxine is a critical-dose drug because a slight change in the blood concentration may result in treatment failure and iatrogenic thyrotoxicosis 6. Furthermore, more than one-third of patients who taked replaced thyroxine remian exhibit persistent symptoms 7. Obviously, deeper investigation should be carried out to determine the underlying mechanisms of HT and to explore more novel potential targets for effective therapies against HT 8.

Pyroptosis is a type of inflammatory programmed cell death mediated by gasdermin D (GSDMD), which is characterized by nod-like receptor protein-3 (NLRP3) inflammasome activation, caspase activation, cell membrane pore formation, and the release of IL-1β 9, 10. Accumulating evidence has revealed that abnormal pyroptosis exists and plays important roles in many kinds of autoimmune diseases 11-13. Recently, Guo *et al.* 14 found that the expression of pyroptosis proteins, such as NLRP3, caspase-1, and pro-IL-1β was significantly increased in thyroid tissues of HT patients, and the thyroid mRNA level of inflammasomes was positively correlated with the contents of TPO-Ab and Tg-Ab in serum. Our previous study suggests that the protein expression of GSDMD and IL-1β is significantly increased in the thyroid tissues of HT patients. In addition, we reveal that excessive iodine increases GSDMD-N and NLRP3 inflammasome activation in human thyroid follicular epithelial cells (TFCs) and promotes IL-1β release *in vitro* 15. Thus, anti-pyroptosis may be a new strategy for the treatment of HT.

It is well established that parasites and eggs from helminths induce host immunosuppression; therefore, they can be used for the remedy of immune disorders 16. SJMHE1 is an HSP60-derived peptide from *Schistosoma japonicum* 17_,_ and our previous studies report that SJMHE1 alleviates collagen-induced arthritis, suppresses delayed-type hypersensitivity and airway inflammation in allergic asthma in murine models 18-20. Importantly, we found that SJMHE1 inhibits pyroptosis-related IL-1β secretion from human peripheral blood mononuclear cells with LPS stimulation *in vitro* 18, suggesting that SJMHE1 might play a regulatory role in excessive iodine-induced abnormal pyroptosis in TFCs. In this study, we investigated the effect and possible associated mechanisms of SJMHE1 on excessive iodine-induced pyroptosis in TFCs.

## Materials and Methods

### Chemicals and Reagents

SJMHE1 437-460 (VPGGGTALLRCIPVLDTLSTKNED) was synthesized and purified by Top-peptide (Shanghai, China) as previously described 18. FSL-1 was purchased from MedChem Express (Monmouth Junction, NJ); Sodium iodide (NaI) was purchased from Sigma-Aldrich (St. Louis, MO); anti-TLR2, anti-TLR4, and IgG isotype control antibodies were purchased from BioLegend (San Diego, CA).

### Cell Culture and Treatment

The thyroid follicular epithelial cell line Nthy-ori 3-1 was purchased from European Collection of Animal Cell Cultures (ECACC). Nthy-ori 3-1 cells were cultured in RPMI-1640 (Biological Industries, Kibbutz Beit Haemek, Israel) containing 10% FBS (Biological Industries) and 2 mM L-glutamine (Biological Industries) and maintained under essential conditions (37 °C, 5% CO_2_). Cells were seeded on 6-well plates and grown until the cell density reached approximately 80%-90% for subsequent experimentation.

Nthy-ori 3-1 in culture was exposed to SJMHE1 at 3 different concentrations (0.1, 0.5 and 1.0 µg/mL). These concentrations were selected based on existing literature on concentrations of SJMHE1 *in vivo* as well as *in vitro* studies regarding the effects of SJMHE1 on other human cells 17. The NaI concentration (50 mM) used to stimulate cells was based on our previous study 15. All treatments were continuously cultured for 24 h.

### Cell Viability Assays

To explore the influences of SJMHE1 and NaI on Nthy-ori 3-1, cell viability was evaluated with Cell Counting Kit-8 (CCK-8, MultiSciences, Hangzhou, China) following the manufacturer's description. Briefly, Nthy-ori 3-1 cells (5 × 10^3^) were plated in a 96-well plate at 100 μL/well and then treated with different concentrations of SJMHE1 (0.1, 0.5 and 1.0 µg/mL) for 24 h. Cells in other wells were treated with NaI (50 mM) with or without SJMHE1 for 24 h. The control for all series was cells exposed to only culture medium for the same amount of time. Then, 10 μl of CCK-8 solution was added to each well and incubated at 37 °C for 2 h. Finally, the absorbance at 450 nm was detected with a microplate reader (Bio-Rad Laboratories, Hercules, CA).

### Flow Cytometry Analysis

Nthy-ori 3-1 cells (3 × 10^5^ cells/well) were cultured on a 6-well plate at 2 mL/well and stimulated with different concentrations of SJMHE1 (0, 0.1, 0.5 and 1.0 µg/mL) for 24 h. The percentage of apoptotic cells was assessed using a FITC Annexin V Apoptosis Detection Kit (Beyotime) in accordance with the manufacturer's instructions.

Reactive oxygen species (ROS) generation was estimated by DCFH-DA (Beyotime). Nthy-ori 3-1 cells were incubated with different concentrations of SJMHE1 (0, 0.5 and 1.0 µg/mL) at a concentration of 50 mM NaI. After 24 h, cells were collected and washed with PBS and then incubated with DCFH-DA for 30 min at 37 °C. After three washes with PBS, DCF fluorescence was emitted by flow cytometry (FACSCanto; BD Biosciences, Franklin Lakes, NJ) using a 488/525 nm excitation/emission filter.

### Western Blotting Analysis

The Western blotting procedure has been described previously 21. After the indicated treatments, Nthy-ori 3-1 cells were collected and lysed by radioimmunoprecipitation assay buffer (Beyotime), and the concentration of the protein was measured using the Bradford assay. Total protein (50 μg) was separated by 10% SDS-PAGE and transferred to PVDF membranes followed by blocking with 5% milk in Tris-buffered saline and 0.5% Tween-20 for 1 h at room temperature. Following blocking, membranes were incubated with primary antibodies overnight at 4°C and then incubated with the anti-mouse or anti-rabbit horseradish peroxidase antibody for 1 h at room temperature. The following antibodies were used: the primary antibody Caspase 3 (9662S), cleaved Caspase 3 (9661S), Caspase 1 (2225S), p38 (8690S), p-p38 (4511S), JNK (9258S), p-JNK (4668S), ERK (4695S), p-ERK (4370S), NF-κB-p65 (8242S), p-NF-κB-p65 (3033S), IκB-α (4814S) and p-IκB-α (9246S) were purchased from Cell Signaling Technology (Danvers, MA); antibody of NLRP3 (ab263899) was bought from Abcam (Cambridge, MA); antibodies against GSDMDC1 (sc-393581), GAPDH (sc-365062), TLR2 (sc-21759), and TLR4 (sc-3072) were purchased from Santa Cruz Biotechnology Inc. The signals of the immunoreactive bands were detected by a chemiluminescence kit (Thermo Fisher Scientific, Waltham, MA). All experiments were repeated three times.

### ELISA for IL-1β Measurements

The level of IL-1β was detected with ELISA kits (MultiSciences, Hangzhou, China). Nthy-ori 3-1 cultures were collected after coculture with SJMHE1 (0, 0.5 and 1.0 µg/mL) and NaI (50 mM), and IL-1β generation was carried out according to the manufacturer's protocols. The absorbance at 450 nm and 630 nm was measured with a microplate reader. The concentrations of IL-1β were calculated with the standard curve.

### Blocking of TLR Pathways with Monoclonal Antibodies

To evaluate whether the SJMHE1-induced Nthy-ori 3-1 effect was dependent on TLR2 or TLR4, Nthy-ori 3-1 cells were preincubated with anti-TLR2 (10 µg/mL), anti-TLR4 (10 µg/mL) or anti-IgG antibodies (10 µg/mL) for 1 h and then treated with NaI (50 mM) with or without SJMHE1 (1.0 µg/mL) for 24 h incubation. The production level of IL-1β and the expression levels of pyroptosis-related proteins, including NLRP3, GSDMD-N and C-caspase-1, were measured as described above.

### Statistical analysis

Statistical analysis was performed using GraphPad Prism 5.0. Student's *t*-test was used to calculate the differences between two groups. Statistical differences between experimental groups were conducted using one-way analysis of variance (ANOVA) with Tukey's multiple-comparison test. Data were all presented as mean ± SD. *P* < 0.05 in different groups was considered to be significant.

## Results

### Effects of SJMHE1 on cell viability and apoptosis

**Figure 1:**
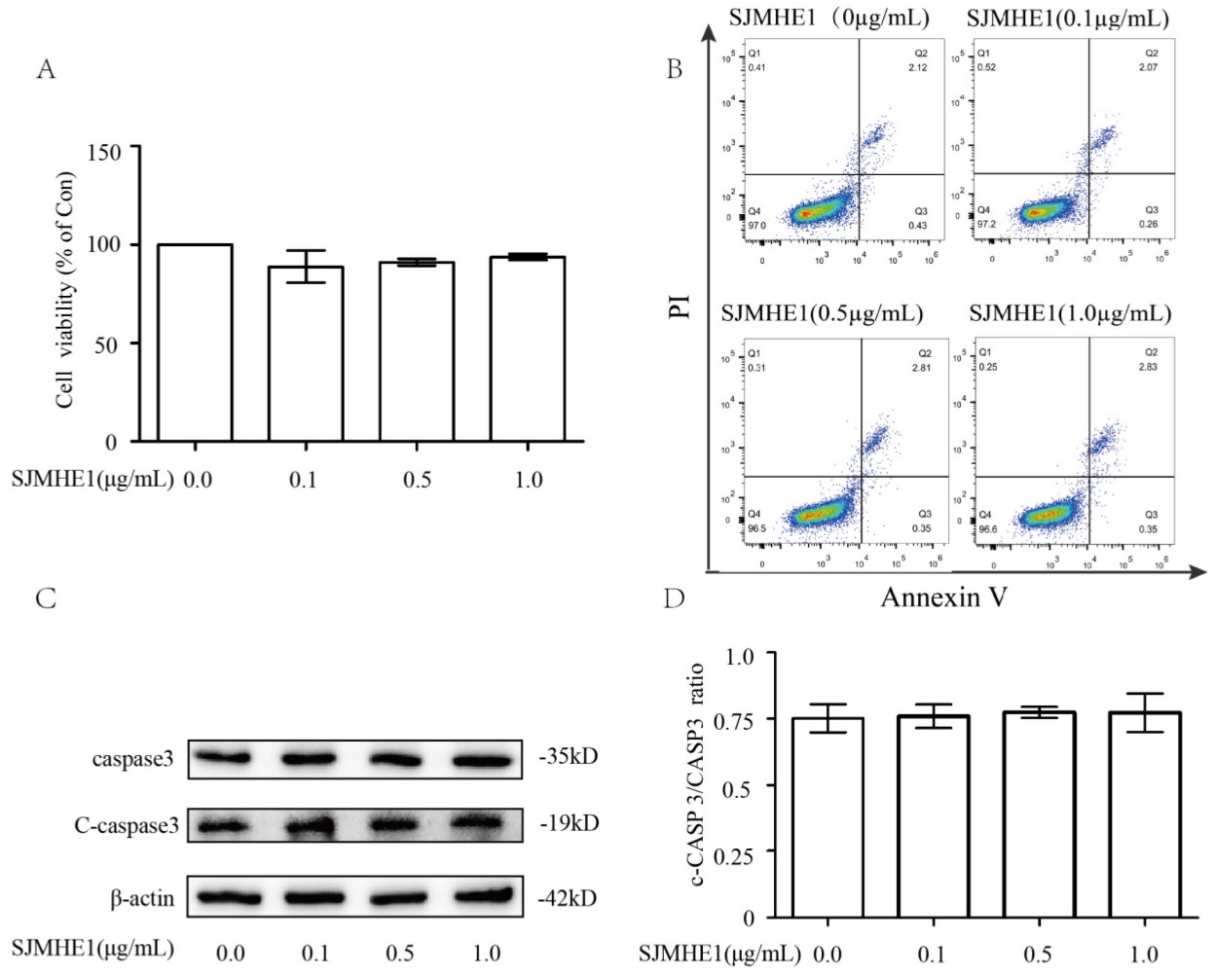
** Cytotoxicity effects of SJMHE1 on TFCs. (A)** Nthy-ori3-1 cells were treated with gradient concentrations of SJMHE1 (0, 0.1, 0.5 and 1.0 µg/mL) for 24 h, and then the viability of Nthy-ori 3-1 cells was evaluated by CCK-8 assay (n = 5).** (B)** After treatment with SJMHE1 at different concentrations for 24 h, apoptosis of Nthy-ori 3-1 cells was determined by flow cytometry (n = 3).** (C)** The protein levels of cleaved Caspase 3 and Caspase 3 in Nthy-ori 3-1 cells treated with 0, 0.1, 0.5 and 1.0 µg/mL SJMHE1 for 24 h were analyzed by Western blotting. The levels of β-actin were used as an internal control. The results shown are representative of three replicates. **(D)** Grayscale analysis was used to quantify the relative expression of Cleaved caspase 3 and caspase 3. Data are expressed as the mean ± SD.

To evaluate the cytotoxiciy of SJMHE1 toward human Nthy-ori 3-1 cells, we examined cell viability using the CCK-8 assay. As shown in Fig. 1A, compared with control (0 µg/mL SJMHE1), none of the gradient concentrations of SJMHE1 (0.1, 0.5 and 1.0 µg/mL) had significant impacts on cell viability. Additionally, treatment of TFCs with SJMHE1 did not alter the percentage of apoptotic cells as measured by flow cytometry for each group (Fig. 1B). To further verify the effect of SJMHE1 on apoptosis, the protein expression levels of caspase 3 and cleaved caspase 3 (C-caspase 3) were detected by Western blotting. The data indicated that C-caspase 3/Caspase 3 ratio was unaffected by challenging the cells with SJMHE1 (Fig. 1C,D). Overall, these results suggested that SJMHE1 did not show any cytotoxicity on Nthy-ori 3-1 at the indicated concentration, therefore, we used 0.5 and 1.0 µg/mL SJMHE1 in the following experiments.

### SJMHE1 protected against excessive iodine-induced pyroptosis in TFCs

**Figure 2:**
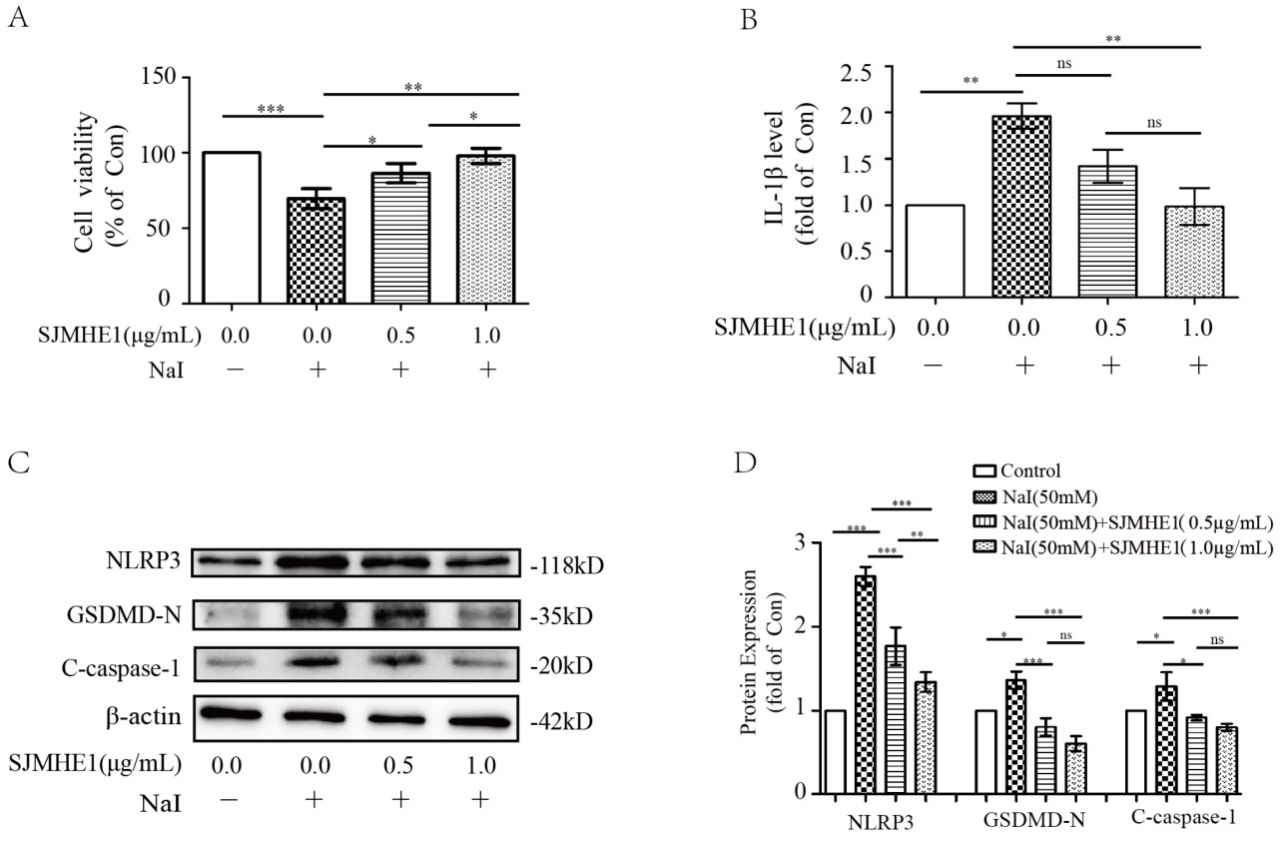
** Antipyroptotic effects of SJMHE1 against excessive iodine-stimulated TFCs.** Nthy-ori 3-1 cells were treated with NaI (50 mM) with gradient concentrations of SJMHE1 (0, 0.5 and 1.0 µg/mL) for 24 h. Control (Con) values were obtained in the absence of NaI. **(A)** The viability of Nthy-ori 3-1 cells was evaluated by CCK-8 assay (n = 5). **(B)** The inflammatory factor IL-1β in the culture supernatants was evaluated by ELISA (n = 3). **(C,D)** Pyroptosis-related proteins, including NLRP3, GSDMD-N and C-caspase-1, were examined by Western blotting. The levels of β-actin were used as an internal control. All values are expressed as the mean ± SD (n = 3 per group). *P < 0.05, **P < 0.01, ***P < 0.001, ns = no significance compared with the involved group.

To test whether SJMHE1 has a protective effect for excessive iodine-induced TFC pyroptosis, Nthy-ori 3-1 cells were treated by 50 mM NaI and were incubated with different doses of SJMHE1 (0.5 and 1.0 µg/mL). As shown in Fig. 2A, Nthy-ori 3-1 cells exhibited significantly elevated viability after treatment with SJMHE1 for 24 h in a dose-dependent manner, compared to the NaI-challenged positive control. Then, we conducted ELISA analysis on cell culture supernatants to investigate whether SJMHE1 could decrease the excessive iodine-induced secretion of IL-1β. As shown in Fig. 2B, treatment with 0.5 and 1.0 µg/mL SJMHE1 for 24 h resulted in a remarkable decrease in NaI-stimulated secretion of extracellular IL-1β. Excessive iodine stimulation also increased the expressions of NLRP3, GSDMD-N and C-caspase-1 proteins in TFCs, in accordance with our previous report 14. Importantly, the NaI-induced upregulations on NLRP3, GSDMD-N and C-caspase-1 were significantly prevented by SJMHE1 treatment in a dose-dependent manner (Fig. 2C,D). Obviously, to a certain extent, these results indicated that SJMHE1 was able to attenuate the excessive iodine-induced pyroptosis in TFCs.

### SJMHE1 inhibits ROS production and attenuates the activation of the MAPK and NF-κB signaling pathways in excessive iodine-stimulated TFCs

**Figure 3:**
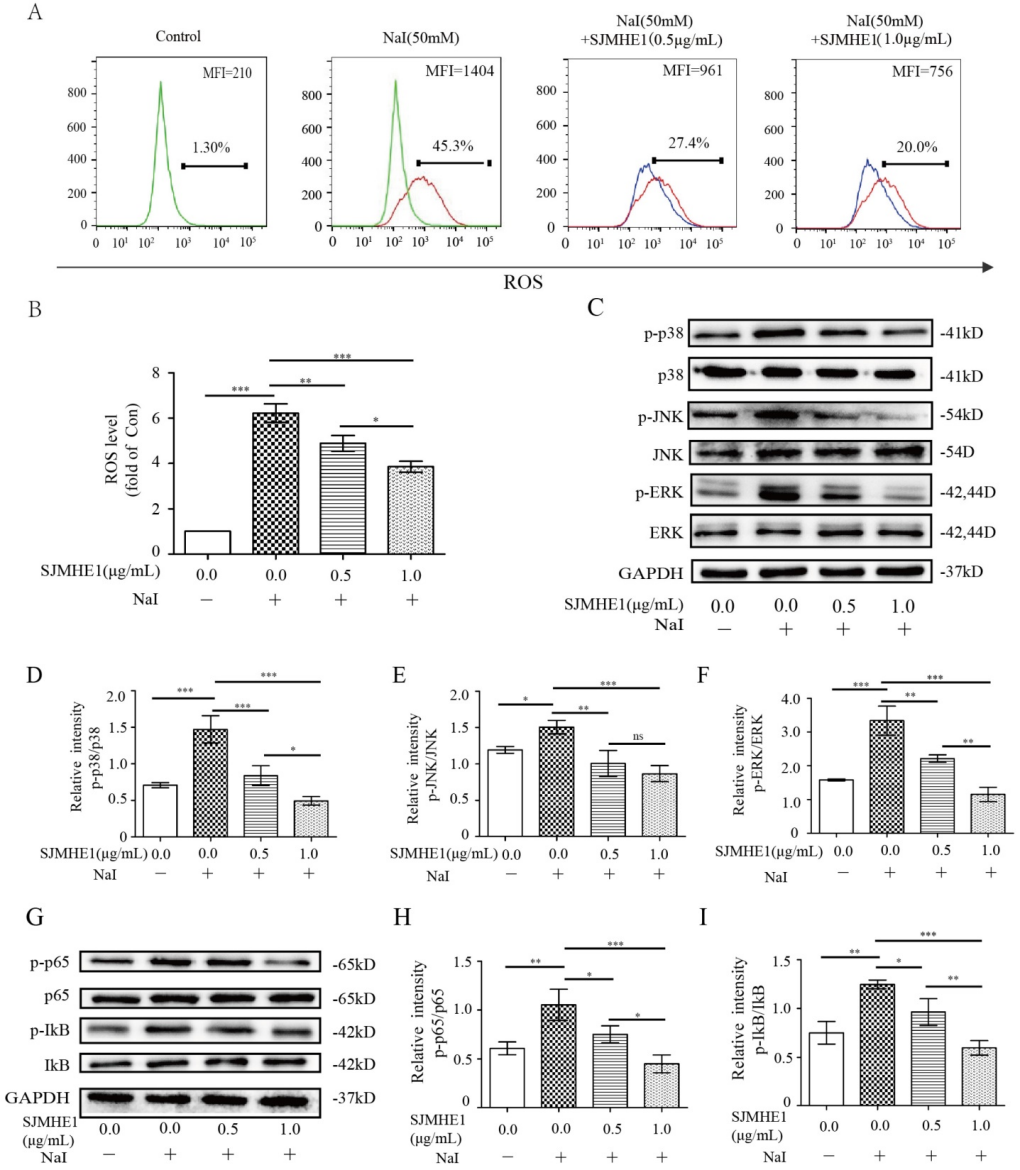
** Roles of SJMHE1 in the generation of ROS and the expression of MAPK/NF-κB signaling pathways in excessive iodine-stimulated TFCs. (A,B)** The mean fluorescence intensity (MFI) of intracellular ROS in Nthy-ori 3-1 cells treated with various concentrations of SJMHE1 (0, 0.5 and 1.0 µg/mL) or 50 mM NaI for 24 h was examined and analyzed by flow cytometry. **(C-F)** The protein levels of p38, JNK, ERK and their phosphorylated forms (p-p38, p-JNK and p-ERK) were detected by Western blotting analysis in Nthy-ori 3-1 cells. **(G-I)** The protein levels of IκB-α, p65 and their phosphorylated forms (p-IκB-α and p-p65) were analyzed by Western blotting in Nthy-ori 3-1 cells. GAPDH was used as the internal standard for normalization. All values are expressed as the mean ± SD (n = 3 per group). *P < 0.05, **P < 0.01, ***P < 0.001 compared with the involved group.

Emerging experimental evidence has showed that both ROS and MAPK/ NF-κB play important roles in the responses to pyroptosis and NLRP3 activation 22-24. Therefore, we further determined the impact of SJMHE1 on the excessive iodine-induced generation of ROS and the protein expression of the MAPK/NF-κB signaling pathway. Nthy-ori 3-1 cells were treated as described above, then analyzed by flow cytometry and Western blotting. As shown in Fig. 3A and B, SJMHE1 administration inhibited the ROS generation in a dose-dependent manner. Moreover, SJMHE1 treatment clearly attenuated excessive iodine-stimulated p38, JNK, ERK1/2 (MAPK pathway) and IκBα, p65 (NF-κB pathway) phosphorylation protein expressions in a dose-dependent manner (Fig. 3C-I). In conclusion, our results suggested that SJMHE1 inhibited ROS production, attenuated the activation of the MAPK and NF-κB signaling pathways in excessive iodine-stimulated TFCs.

### SJMHE1 exerts anti-pyroptotic effects in excessive iodine-induced TFCs by targeting TLR2 but not TLR4

**Figure 4:**
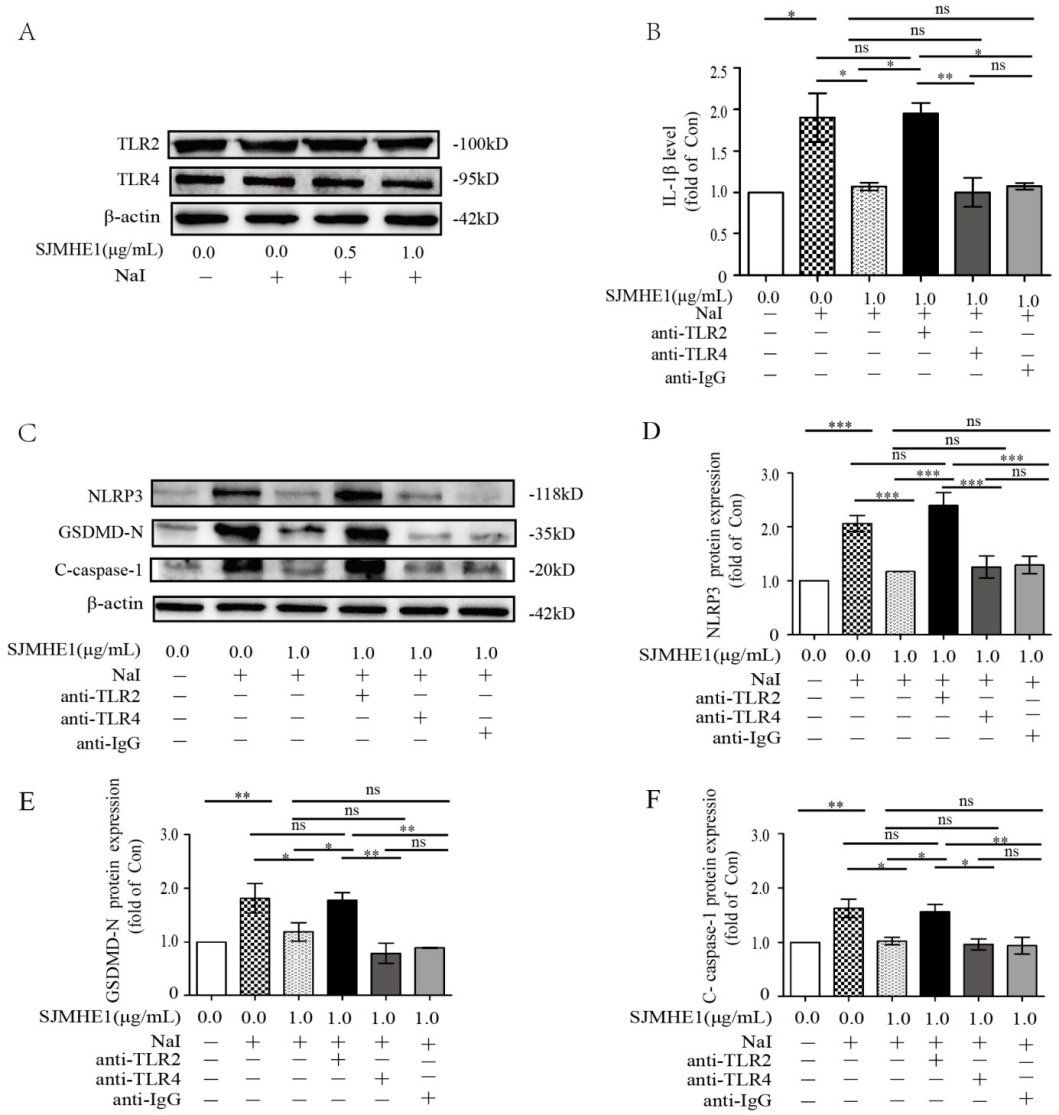
** Participation of TLR2 but not TLR4 in SJMHE1 induced an anti-pyroptosis process in excessive iodine-stimulated TFCs. (A)** TLR2 and TLR4 expression in Nthy-ori 3-1 cells treated with the indicated concentration of SJMHE1 was evaluated in the presence of NaI (50 mM) for 24 h using Western blotting. B:Nthy-ori 3-1 cells were pretreated with blocking antibodies (TLR2, TLR4 and IgG) before stimulation with NaI (50 mM) and SJMHE1 (1 µg/mL). **(B)** ELISA was performed to measure the levels of IL-1β. **(C-F)** Western blotting analysis was performed to analyze the levels of NLRP3, GSDMD-N and C-caspase-1. The levels of β-actin were used as an internal control. The relative band intensity of each protein was analyzed by ImageJ. All values are expressed as the mean ± SD (n = 3 per group). *P < 0.05, **P < 0.01, ***P < 0.001, ns = no significance compared with the involved group.

Helminth-derived products have evolved to mediate their anti-inflammatory effects through major pattern recognition receptors 25. SJMHE1 is an HSP60-derived peptide from Schistosoma japonicum, and studies have demonstrated that HSP60 is a natural ligand for TLR2 and TLR4 26. Therefore, we further investigated whether TLR2 or TLR4 are involved in SJMHE1-mediated pyroptosis inhibition. Nthy-ori 3-1 cells expressed TLR2 and TLR4, and their expression was unaffected by the stimulation of excessive iodine (50 mM NaI) or SJMHE1 (0.5 and 1.0 μg/mL), respectively (Fig. 4A). Blocking of the TLR2 or TLR4 with corresponding monoclonal antibodies, then co-treatment with SJMHE1 and excessive iodine in Nthy-ori 3-1 cells, the IL-1β release and expressions of NLRP3, GSDMD-N and C-caspase-1 were examined by using ELISA and Western blotting, respectively. As shown in Fig. 4B, Nthy-ori 3-1 cells incubated with SJMHE1 and co-treated with anti-TLR2 antibodies, but not anti-TLR4 antibodies, showed a visible increase in the level of IL-1β compared to the SJMHE1-treated alone group. Not only inflammatory IL-1β proteins, but also NLRP3, GSDMD-N and C-caspase-1 expressions showed similar results (Fig. 4C-F). These findings partly suggested that SJMHE1 is a TLR2 ligand. In addition, as shown in Supplementary Fig. 1, FSL-1 (TLR2 agonist) increased the protein level of p-p65 (marker of TLR2 signal activation) in Nthy-ori 3-1 cells, compared with that of the control group. While, compare to control group, the protein level of p-p65 was suppressed with SJMHE1 treatment. Moreover, compared to FSL-1 group, co-treatment with FSL-1 and SJMHE1 resulted in the decrease of p-p65 protein expression, suggesting SJMHE1 might not be a TLR2-agonist, but a TLR2 signal inhibitor. Taken together, these findings suggested that SJMHE1 might play a suppressive role in excessive iodine-induced TFC pyroptosis through the inhibitory manipulation of TLR2 signaling.

## Discussion

TFC pyroptosis, which mediates the release of IL-1β and is link to the increase in contents of TPO-Ab and Tg-Ab in serum, is involved in the pathogenesis of HT 14, 15. Therefore, TFC pyroptosis may represent a potential therapeutic target of HT. In this study, we showed that the *Schistosoma japonicum* peptide SJMHE1 inhibits excessive iodine-induced pyroptosis of TFC cells.

After the hygiene hypothesis was proposed, helminths (especially excretory/secretory) have emerged as novel sources of therapeutics for dysregulated inflammatory diseases 27. Although helminth-derived molecules are thought to offer protection against the development of autoimmune diseases 28, most immunomodulatory regents that have been identified thus far, such as soluble egg antigen, are mixtures and macromolecules with potential cytotoxicity 29. SJMHE1 is a small peptide from the HSP60 protein of S. japonicum. We previously showed that the cell viability of RAW264.7 cells is not affected by SJMHE1 30. Similarly, in the present study, we found that SJMHE1 does not exert cytotoxicity to Nthy-ori 3-1, further suggesting that SJMHE1 may be a safe selection of small molecules from helminths.

Pyroptosis is a form of programmed cell death accompanied by inflammatory responses 31. The classical pyroptosis pathway depends on caspase-1, which is mediated by NLRP3 recruits and caspase-1 activation. C-caspase-1 then cleaves GSDMD to form GSDMD-N, causing cell membrane perforation, leading to reduction in cell viability and the release of IL-1β 32. SJMHE1 inhibits IL-1β secretion from human peripheral blood mononuclear cells with LPS stimulation *in vitro* 18. In the present study, we found that SJMHE1 decreased the content of IL-1β in the cell supernatant from excessive iodine-induced TFC. Furthermore, we observed that SJMHE1 treatment unregulated cell viability and decreased protein levels of NLRP3, GSDMD-N, and C-caspase-1 in excessive iodine-treated TFC, indicating that SJMHE1 has a certain role in anti classical pyroptosis.

Consistent with previous reports 15, 33, 34, excessive iodine substantially increased the generation of ROS and the activation of MAPK/NF-κB signaling in TFCs, indicating that the ROS-NF-κB-NLRP3 and MAPK pathways are involved in TFC pyroptosis. Interestingly, we found that SJMHE1 treatment dramatically inhibited ROS generation and MAPK/NF-κB activation in excessive iodine-treated TFCs, further confirming that SJMHE1 has inhibitory effects on TFC pyroptosis from the molecular point of view.

**Figure 5:**
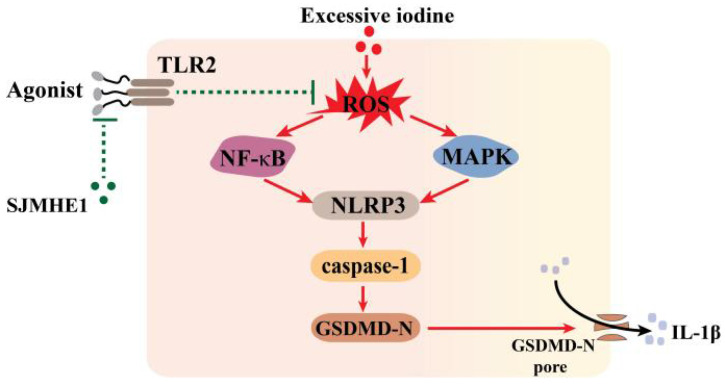
Schematic diagram showing the effect of SJMHE1 on excessive iodine-induced pyroptosis in TFCs.

Helminth-derived products exert their anti-inflammatory effects through pattern recognition receptors 25. TLR2-blocking antibodies significantly attenuated the inhibitory effects of SJMHE1 on excessive iodine-induced pyroptosis, suggesting that SJMHE1 needs to conjugate TLR2 to exert function, which consistent with our previous study that SJMHE1 induces Tregs by converting non-Tregs into Tregs via TLR2 17. Harnett and colleagues reported that helminth-derived ES-62 has an inhibitory effect on its receptor signal (TLR4), but could not alter the surface expression of TLR4-MD-2 35, 36. We found that SJMHE1 could not affect the protein level of TLR2 in TFCs, while antagonized TLR2-signaling induced by FSL-1. In addition, our results showed there was a degree of spontaneous p65 activation in TFCs, and which was also quenched by SJMHE1, suggesting SJMHE1 might block the constitutive TLR2 activation. Thus, we speculated that the mechanism of SJMHE1 interfering with excessive iodine-induced pyroptosis was accomplished through two different signaling pathway and intracellular cross-talk interaction, which resulted in an inhibition of ROS generation (Fig. 5). However, some unsolved questions would still remain in present study, such as what causes the spontaneous TLR2 activation, and how does the TLR2 antibody selectively block the SJMHE1 without interfering with the background activation. In addition, the mechanisms by which SJMHE1 inhibits the TLR2 activation remain unclear. Importantly, further studies are warranted to evaluate the anti-pyroptotic effects of SJMHE1 in HT mouse model.

In conclusion, our study demonstrated that SJMHE1 exerts anti-pyroptotic activity in excessive iodine-induced TFCs via a TLR2-dependent manner and may be a promising candidate target for preventing HT.

## Supplementary Material

Supplementary figure.

## Abbreviations

HT: Hashimoto's thyroiditis
GSDMD-N: N-terminus of gasdermin D
NaI: Sodium iodide
NLRP3: nod-like receptor protein-3
ROS: Reactive oxygen species
SJMHE1: Schistosoma japonicum peptide
TFCs: thyroid follicular epithelial cells
TLR: Toll-like receptor
