# Supplementary material for: SJMHE1 protects against excessive iodine-induced pyroptosis in human thyroid follicular epithelial cells through a toll-like receptor 2-dependent pathway
Source: Int J Med Sci. 2022 Mar 21;19(4):631–9. doi: 10.7150/ijms.66167 (PMC9108411; doi:10.7150/ijms.66167)
Supplement: Supplementary file 1 — Supplementary figure. [file ijmsv19p0631s1.pdf]

Supplementary

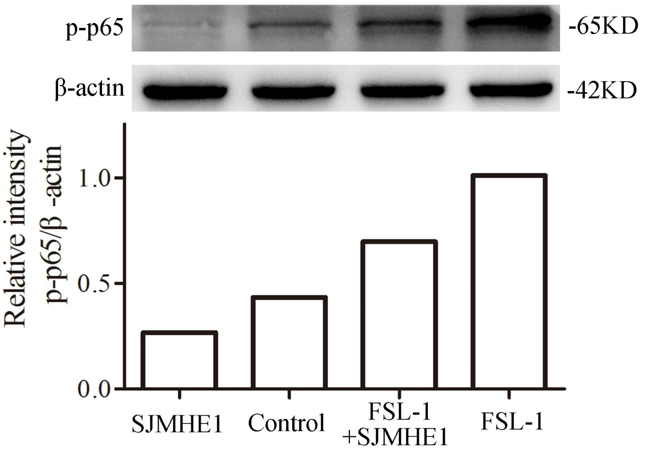

Supplementary Fig. SJMHE1 inhibits FSL-1-induced activation of Nthy-ori 3-1 cells. Western blot analyses the inhibitory effects of SJMHE1 on FSL-1-induced NF-κB (a representative's result was showed from two independent experiments).
